# Supplementary material for: Rapid and simultaneous detection of Escherichia coli and Klebsiella pneumoniae: a novel dual recombinase polymerase amplification-clustered regularly interspaced short palindromic repeats-Cas12a method
Source: Microbiol Spectr. 2026 Mar 30;14(5):e03598-25. doi: 10.1128/spectrum.03598-25 (PMC13142036; doi:10.1128/spectrum.03598-25)
Supplement: Supplemental material — Tables S1 to S5. [file spectrum.03598-25-s0001.docx]

| **Name** | **Category number** | **Source** |
| --- | --- | --- |
| *P.aeruginosa* | ATCC27853 | American Type Cultur Collection |
| *K.pneumoniae* | ATCC700603 | American Type Cultur Collection |
| *S.aureus* | ATCC29213 | American Type Cultur Collection |
| *E.faecalis* | ATCC29212 | American Type Cultur Collection |
| *E.coli* | ATCC35218 | American Type Cultur Collection |
| *E.faecium* | GDMCC1.388 | Guangdong Microbial Culture Collection Center |
| *S.aureus* | ATCCBAA1026 | American Type Cultur Collection |
| *S. pneumoniae* | ATCC49619 | American Type Cultur Collection |
| *H. haemolyticus* | ATCC33390 | American Type Cultur Collection |
| *M. catarrhalis* | - | Clinical isolates |
| *S. maltophilia* | - | Clinical isolates |
| *E. cloacae* | - | Clinical isolates |
| *A. baumannii* | - | Clinical isolates |

**Table S1. Information on pathogenic microorganisms in this study**

**Table S2. Sequences of primers, crRNA, and ssDNA reporters in this study**

| **Primer** | **Base sequences (5'-3')** | **Base number** |
| --- | --- | --- |
| uidA-primer1 | F:CTTACTTCCATGATTTCTTTAATTATGCCG | 30 |
|  | R:GACGCACAGTTCATAGAGATAACCTTCA | 28 |
| uidA-primer2 | F:AAGCAGTCTTACTTCCATGATTTCTTTAAT | 30 |
|  | R:TGACGCACAGTTCATAGAGATAACCTTC | 28 |
| uidA-primer3 | F:ATTTCTTTAATTATGCCGGGATCCATCG | 28 |
|  | R:TGACGCACAGTTCATAGAGATAACCTTC | 28 |
| uidA-primer4 | F:AGAAAAAGCAGTCTTACTTCCATGATTTC | 29 |
|  | R:TGACGCACAGTTCATAGAGATAACCTTC | 28 |
| rcsA-primer1 | F:TGGATTTGTGCAGCTATACCCGGTTGGGATTG | 32 |
|  | R:CGCAAATAGCGGTCAAAATGGATGTTCGCCAG | 32 |
| rcsA-primer2 | F:AATCTGCTAATCAGTTCAAAATCGATAAC | 29 |
|  | R:ATAAATCACCTGCTTATTATGCGTTTGTAT | 30 |
| rcsA-primer3 | F:ATCTGCTAATCAGTTCAAAATCGATAACCC | 30 |
|  | R:GATAAATCACCTGCTTATTATGCGTTTGTA | 30 |
| rcsA-primer4 | F:CTTTCTAAACCTACTATTATTATCGCCCGC | 30 |
|  | R:GAAACAGTCTTCATTCAGAAACACCACC | 28 |
| rcsA-primer5 | F:ATCTTAAATACAAAAACACCAGTGTAGGG | 29 |
|  | R:TATGATAAATCACCTGCTTATTATGCGTT | 29 |
| rcsA-primer6 | F:CCAAAAGACCTTGATGTTATTCTGGTTAAT | 30 |
|  | R:TATGATAAATCACCTGCTTATTATGCGTTT | 30 |
| rcsA-crRNA1 | UAAUUUCUACUAAGUGUAGAUCGAAGUACCAUGCCCGGCCAU | 42 |
| rcsA-crRNA2 | UAAUUUCUACUAAGUGUAGAUAUGUUCAUUUGCGUUGAGAUU | 42 |
| uidA-crRNA1 | UAAUUUCUACUAAGUGUAGAUAUUAUGCCGGGAUCCAUCGCA | 42 |
| uidA-crRNA2 | UAAUUUCUACUAAGUGUAGAUCAAGUGGUGAAUCCACACCUC | 42 |
| SSDNA | FAM-TTATT-BHQ1 | 5 |
| PCR-uidA | F:ATGCGGATCAACAGGTGGTT  R:TCTGTCTGGCTTTTGGCTGT | 20 |
| PCR-rcsA | F:ACGTGTCGATTGAGGATGGG  R:ATCCGCAGCATTGTTGACCT | 20 |

**Table S3. Reproducibility of the dual RPA-CRISPR/Cas12a method**

| **Gene** | **RFU** | $\bar{\text{x}}$ | **SD** | **RSD** |
| --- | --- | --- | --- | --- |
| uidA | 5021.00 |  |  |  |
|  | 5016.00 |  |  |  |
|  | 5394.00 | 5205.8 | 195.07 | 3.75％ |
|  | 5420.00 |  |  |  |
|  | 5178.00 |  |  |  |
| rcsA | 8487.00 |  |  |  |
|  | 8241.00 |  |  |  |
|  | 8958.00 | 8724.2 | 334.93 | 3.84％ |
|  | 8746.00 |  |  |  |
|  | 9189.00 |  |  |  |

**Table S4. Comparison of different detection methods**

| **Detection method** | **Amplification method** | **Temperature** | **Number of primers** | **Sensitivity** | **Detection time** |
| --- | --- | --- | --- | --- | --- |
| PCR-CRISPR/Cas12a | RPC | 94℃-58℃-72℃ | 2 | 92% | >2 h |
| LAMP-CRISPR/Cas12a | LAMP | 65℃ | 6 | 9.2 CFU/mL | 1 h |
| Dual RPA-LFD method | dual RPA | 37℃ | 2 | ST(52.3 CFU/mL)  SE(35.9 CFU/mL) | 65 min |
| RCA-CRISPR/Cas12a | RCA | 37℃ | 2 | 53 copies/μL | 10 min |
| RAA-TS | RAA | 37℃ | 2 | gyrB(50 CFU/mL）  aprX(250 CFU/mL | 90 min |
| HhaI -glyceryl-RPA-Cas12a | RAP | 37℃ | 2 | 100aM | 70 min |
| Dual RPA-CRISPR/Cas12a（This study） | dual RPA | 37℃ | 2 | uidA(53.7 copies/μL)  rcsA(59 copies/μL) | 70 min |

**Table S5. Dual-RPA reaction system**

| **constituents** | **volume（μL）** | **concentration** |
| --- | --- | --- |
| Premix (containing enzymes, buffer solution, enzyme-free water, primers) | 8.7 | - |
| *K. pneumoniae* (rcsA) *DNA template* | 0.4 | - |
| *E. coli (uidA) DNA template* | 0.4 | - |
| MgOAc | 0.5 | 14mM |
| Total volume of a single reaction | 10 | - |
| Premix |  |  |
| Basic buffer | 5.9 | - |
| *K. pneumoniae* F/R primers | 0.48/ 0.48 | 480nM |
| *E. coli* F/R primers | 0.48 /0.48 | 480nM |
| enzyme-free water | 0.88 | - |
| Total volume of the single-component premix | 8.7 |  |
